# Neuroepithelial Cell Transforming Gene 1 Acts as an Oncogene and Is Mediated by miR-22 in Human Non-Small-Cell Lung Cancer

**DOI:** 10.1155/2020/1648419

**Published:** 2020-01-11

**Authors:** Shengguang Ding, Haitao Huang, Yiming Xu, Liang Shen, Chongjun Zhong, Shiying Zheng

**Affiliations:** ^1^Department of Thoracic Surgery, The First Affiliated Hospital of Soochow University, Suzhou 215006, China; ^2^Department of Thoracic and Cardiovascular Surgery, The Second Affiliated Hospital of Nantong University, Nantong 226001, China

## Abstract

Abnormal expression of neuroepithelial cell transforming gene 1 (NET1) has been authenticated in many human cancers, including lung cancer. We have previously reported that NET1 functioned as an oncogene and promoted human non-small-cell lung cancer (NSCLC) growth and migration. However, the correlation between NET1 and its upstream miRNAs needed further illustration. Our present work demonstrated that miR-22 had a relatively low expression, and NET1 had a relatively high expression in both NSCLC samples and lung adenocarcinoma cell lines compared with corresponding normal controls. Moreover, miR-22 directly regulated NET1 and was verified to weaken cancer cell proliferation and migration, as well as enhance cell apoptosis by suppressing NET1. Furthermore, the inhibitory effect of miR-22 can be reversed via overexpressing NET1 using an ectopic expression vector in NSCLC cells. Our findings showed that miR-22/NET-1 axis may contribute to the inhibition of NSCLC growth and migration and represents a promising therapeutic target for NSCLC.

## 1. Introduction

Non-small-cell lung cancer (NSCLC) that accounts for 85% of all human lung cancer cases in adults is a leading cause of cancer-associated morbidity and mortality worldwide [1, 2]. However, using low-dose computed tomographic screening to detect lung cancer has been found to decrease mortality of lung cancer patients in USA [3]. Additionally, the present therapeutic strategies for lung cancer, including surgery, radiotherapy, chemotherapy, and molecular targeted therapy, were widely applied for a comprehensive treatment and have greatly improved the survival of patients with NSCLC. The overall prognosis of these patients remains relatively poor [4]. Thus, it is extremely necessary to look for innovative therapeutic strategies for this deadly disease.

The neuroepithelial cell transforming gene 1 (NET1), a RhoA guanine nucleotide exchange factor, was firstly identified as an oncogene in NIH3T3 cells [5]. NET1 functions as a novel regulator of mitosis and occurs in human cancers [6]. In malignant tumors of digestive system, NET1 acts as a driver of tumor cell migration and invasion, an activity facilitated by regulating RhoA and cytoskeletal reorganization in gastric cancer [7]. NET1 expression is upregulated in esophageal cancer (OAC) and Barrett's oesophagus. NET1 enhances OAC cell proliferation and invasion, and it regulates LPA-induced OAC cell migration [8]. NET1 relates to proliferation, metastasis, and TMN stages of hepatocellular carcinoma (HCC) and can promote the progression of HCC by modulating the PI3K/AKT signaling [9, 10]. Meanwhile, NET1 is also an indicator of poor prognosis in HCC and adenocarcinoma of the oesophagogastric junction [11, 12]. Additionally, NET1 increases cell migration in both ER-positive and ER-negative cells [13] and enhances proliferation and chemoresistance and was regulated by miR-206 in B-acute lymphoblastic leukemia (B-ALL) cells [14]. More importantly, NET1 is differently expressed in human NSCLC and can be utilized as a predictor as well as a novel therapeutic approach in NSCLC [15]. Our previous work illustrated that NET1 plays an important role in regulating cellular proliferation and migration of NSCLC via activating the RhoA pathway [16]. However, it is still unknown whether and how NET1 was modulated by miRs in lung cancer.

MicroRNAs (miRNAs) are a group of small noncoding RNAs with about 20–22 nucleotides in length that have emerged as important modulators at posttranscriptional level through degradation of transcripts or translational inhibition mainly via binding to 3′-untranslated region (3′-UTR) of target mRNA [17]. The aberrant expressions of miRNAs are closely associated with various human malignancies by regulating pathophysiological processes, including cell growth, invasion, migration, and apoptosis [18, 19]. As reported, multiple miRNAs such as miR-1254, -361-5p, and -222 have been demonstrated to be involved in the initiation and development of lung cancer [20–23]. Moreover, miR-22 was reduced in both lung cancer tissues and cell lines [24]. miR-22 was upregulated in whole blood and represented a novel predictive biomarker for pemetrexed-based treatment [25]. However, the functional roles and cellular mechanisms of miR-22 in regulating pathophysiological processes of NSCLC are poorly elucidated. Besides, neuroepithelial cell transforming gene 1 is a potential target gene of miR-22 reported in other two types of tumors [26, 27]; however, its biological relationship with miR-22 in human lung cancer remains unclear.

Therefore, we aimed at investigating the functional role of miR-22 in regulating the growth, migration, and apoptosis of human NSCLC cell lines. Our work presents that miR-22 is sufficient to reduce the proliferation and migration and increase apoptosis in lung adenocarcinoma cell lines while suppression of miR-22 has inverse effects. NET1 is a target gene responsible for the effects of miR-22 in NSCLC cell lines.

## 2. Materials and Methods

### 2.1. Tissue Samples and Ethics Statement

All the clinical tissues and corresponding adjacent nontumorous lung samples were enrolled from 30 patients who underwent operations and were diagnosed with lung adenocarcinoma after surgical section. All tissues were immediately frozen in liquid nitrogen immediately and stored at 80°C for further total RNA extraction. The present work was approved by the Ethics Committee of the Second Affiliated hospital of Nantong University (Nantong, China). Signed informed consent was obtained from all enrolled patients.

### 2.2. Cell Culture and Transfection

NSCLC cell lines (A549 and H1299) and normal lung epithelial cells (BEAS-2B cells) were both purchased from the Cell Bank of the Chinese Academy of Sciences (Shanghai, China). The cells were cultivated in in an RPMI 1640 medium supplemented with 10% fetal calf serum (FBS, Gibco, Life Technologies, USA) and 1% penicillin/streptomycin (Invitrogen, Carlsbad, USA) in a humidified atmosphere of 5% CO_2_ at 37°C. The miR-22 mimics, miR-22 inhibitors, and their negative controls of nc-mimic and nc-inhibitor were obtained from RiboBio (Guangzhou, China) and were transfected with miR-22 mimics (50 nM), inhibitors (100 nM), or their negative controls for 48 h using Lipofectamine 2000 (Invitrogen, USA) according to the manufacturer's protocols.

### 2.3. Cell Counting Kit-8 and EdU Incorporation Assays

The effects of miR-22 on the viability of NSCLC cells were measured using cell counting kit-8 assays (CCK-8; Dojindo, Japan). Cells were planted in 96-well plates and were allowed to adhere overnight. After transfection of miR-22 mimics or inhibitors, CCK-8 reagent was added and incubated for 1 h at 37°C, and the absorbance was performed at 450 nm using a spectrophotometer (Bio-Rad, USA).

Cells were seeded in 24-well plates and were allowed to adhere overnight in complete medium. After 48 h of transfection with the miR-22 mimics and their negative control, cells were incubated with EdU (5-ethynyl-2-deoxyuridine) for 8 hours before staining. The actively proliferating cells were then detected using a CellLight™ EdU Cell Proliferation Detection Kit (RiboBio, China) following the manufacturer's instructions. Percentage of proliferative cells was observed with an epifluorescence microscope (Leica, Germany).

### 2.4. Cell Migration Assays

Cell migration of NSCLC cells was evaluated by wound healing assay. In brief, cells were seeded in a 24-well plate with the same numbers in complete medium, respectively, and were incubated overnight. After forming the monolayer, we used a 200-*μ*l pipette tip to scratch a wound by a line each well. Then, we washed the plates with PBS and removed the detached cells, and the remaining cells were cultured in serum free DMEM medium. Pictures of the scratch wounds were subsequently captured at 0 and 48 h. The closure of the wounds was determined by the width of the scratched area.

### 2.5. Luciferase Activity Assay

A fragment of the 3′untranslated region (3′UTR) of the wild-type (WT) or mutant (MUT) NET1 containing the target site of miR-22 was synthesized and then cloned into the pGL3-Basic Vector (Promega). HEK-293T cells were co-transfected with PGL3-basic-3′UTR or PGL3-basic-3′UTR mut, Renila, miR-22 mimic, or negative control using Lipofectamine 2000 Reagent for 24 h. Luciferase activity was measured by a dual-luciferase reporter assay kit (Promega).

### 2.6. Quantitative Reverse Transcription Polymerase Chain Reactions

Total RNA was isolated from cells and tissues using Trizol reagent (TaKaRa) according to the manufacturer's protocols. For quantitative miRNA analysis, total RNA was reverse transcribed to cDNA using iScript™ cDNA Synthesis Kit (Bio-Rad). The Bulge-Loop™ miRNA qPCR Primer Set (RiboBio) was used to confirm the expression level of miR-22 with Takara SYBR Premix Ex Taq™ (TliRNaseH Plus) in CFX96™ Real-Time PCR Detection System. U6 was used as an internal control for miRNA template normalization. The relative expression level for each miRNA was calculated using the 2-ΔΔCt method.

### 2.7. Western Blot Analysis

Tissues and Cells were lysed in RIPA buffer (Beyotime Institute of Biotechnology, China) containing PMSF and protease inhibitor cocktails (Sigma). Equal amounts of protein were subjected to SDS–PAGE gels and then transferred onto a PVDF membrane. The membranes were blocked in TBST with 2% BSA for 2 h and cultured overnight in 4°C with antibodies against NET1 primary antibody (Abcam, 1 : 1000), anti-vimentin (Abcam, 1 : 1000), anti-E-cadherin (Abcam, 1 : 1000), F-actin (Abcam, 1 : 500), BAX(CST, 1 : 1000), and Bcl-2 (CST, 1 : 1000). *β*-Actin (Bioworld, dilution of 1 : 5000) was used as an internal loading control. The blots were detected using HRP-linked secondary antibodies and the ECL System, and all experiments were performed triple times.

### 2.8. Statistical Analysis

All the quantified data are presented as the mean ±SEM and performed in triplicate, and a Student's *t*-test or one-way ANOVA was used to calculate all data via SPSS version 20. *P* value <0.05 was considered as statistically significant. The graphs were made using GraphPad Prism 5.0 software for Windows.

## 3. Results

### 3.1. miR-22 Is Significantly Downregulated in Both NSCLC Tissues and NSCLC Cell Lines

**Figure 1:**
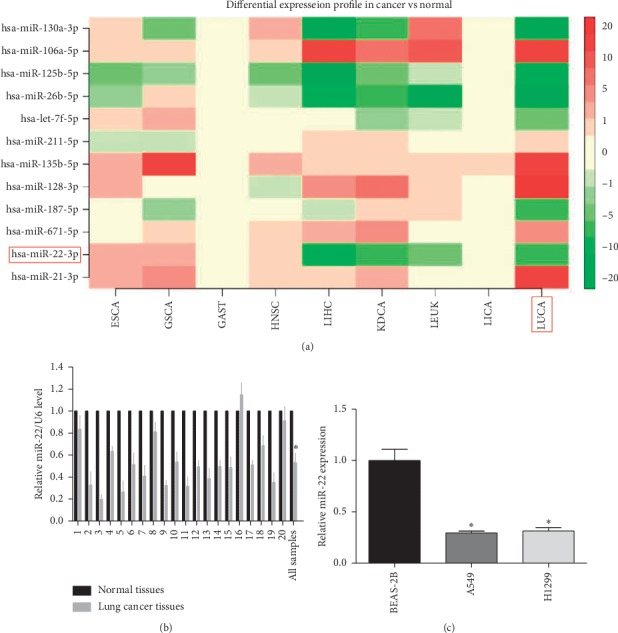
miR-22 is downregulated in human lung cancer. (a) We sought the integrated database for miR-22 expression in normal and cancer tissues using dbDEMC (database of Differentially Expressed MiRNAs in human Cancers) and found that miR-22 was identified to be decreased in human lung cancer compared to normal samples. (b) Quantitative analysis for miR-22 expression standardized against those of U6 using qRT-PCR in normal human lung tissues and in lung tumor tissues (*n* = 10). (c) A qRT-PCR assay was performed to confirm the miR-22 level in NSCLC cell lines and their corresponding control, normalized against U6 (*n* = 3). ^*∗*^*P* < 0.05.

To validate the expression level of miR-22 in human NSCLC tissues, we firstly evaluated miR-22 expression in NSCLC tumor tissues and corresponding adjacent normal tissues provided by searching an integrated database, dbDEMC (database of Differentially Expressed MiRNAs in human Cancers) (http://www.picb.ac.cn/dbDEMC/index.html), and we found that miR-22 was significantly reduced in human NSCLC compared with the normal lung tissues (Figure 1(a)). Then, we further confirmed that miR-22 had a lower expression levels in NSCLC tissues than in-paired adjacent nontumor tissues (Figure 1(b)). Next, miR-22 expression in NSCLC cell lines including A549 and H1299 cells was remarkably decreased in comparison to normal lung epithelial cells (BEAS-2B) (Figure 1(c)). These data represent that miR-22 may play a tumor suppressor role in NSCLC progression.

### 3.2. NET1 Acts as an Oncogene and Is Directly Inhibited by miR-22

**Figure 2:**
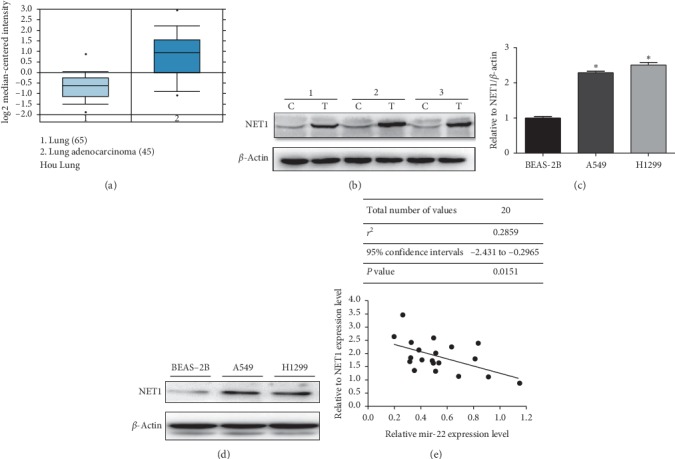
NET1 was significantly increased in both LUAD tumors and LUAD cell lines. (a) We performed to analyze NET1 expression in NSCLC tumor and adjacent normal tissues consulted by Oncomine, derived from Hou Lung. (b) NET1 is remarkably elevated in human LUAD comparison to adjacent normal tissues via using western blot assay (*n* = 4). (c, d) NET1 expression in NSCLC cell lines (A549, H1299) and in immortalized normal lung epithelial cells BEAS-2B as detected by qRT-PCR and western bolting. (e) The related expression between miR-22 and NET1 in 20 paired NSCLC tissues. *β*-Actin and U6 acted as internal controls separately (*n* = 3). ^*∗*^*P* < 0.05.

We reestimated the expression of NET1 in human lung cancer cohort from the Oncomine Platform (https://www.oncomine.org/resource/login.html). The results showed that NET1 was overexpressed in tumor tissues compared with normal tissues derived from Hou Lung (Figure 2(a)). Consistent with this finding, NET1 was highly expressed in the mRNA level of A549 and H1299 cell lines by using QRT-PCR (Figure 2(b)). We then detected NET1 protein levels in lung cancer and adjacent normal tissues, as well as in NSCLC cell lines by western blot. As shown in Figures 2(c) and 2(d), the expression of NET1 was higher in human lung cancers than that in normal controls. Moreover, the Pearson relation showed that the expression level of NET1 in the analyzed NSCLC samples was inversely associated with miR-22 (Figure 2(e)).

**Figure 3:**
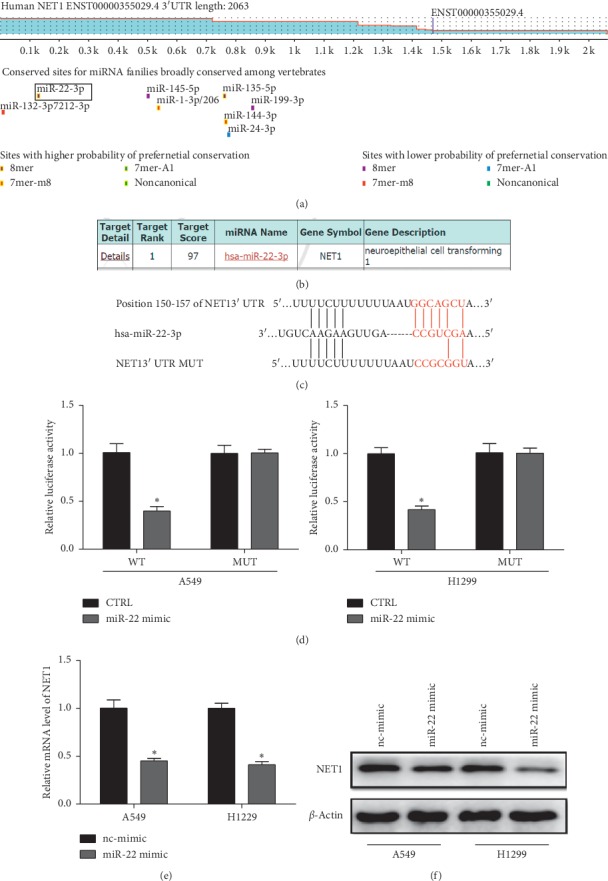
NET1 was directly suppressed by miR-22. (a, b) TargetScan and miRDB bioinformatics analysis revealed that NET1 is predicted to be a target of miR-22. (c) Predicted miR-22 target sequences in the wild-type and mutant 3′UTR regions of NET1. (d) The Luciferase activity assay illustrated that overexpression of miR-22 could decrease the intensity of the fluorescence in both A549 and H1299 cells transfected with the NET1 3′UTR WT vector, while it was invalid with the NET1 3′UTR MUT vector (*n* = 6). (e, f) The expression of NET1 in both mRNA and protein in NSCLC cell lines was significantly reduced following upregulation of miR-22, which was determined by qRT-PCR and western blotting. *β*-Actin was used as a loading control. (*n* = 3). ^*∗*^*P* < 0.05.

To validate the possibility of posttranscriptional function of NET1 modulated by miRNA, we used the bioinformatics tool Targetscan (https://www.targetscan.org) (Figure 3(a)) and miRDB (https://www.mirdb.org) (Figure 3(b)) to predict the latent miRNA targeting NET1. As a result, miR-22 was identified the most optimal miRNA and yielded a notable and consistent decline in the two lung adenocarcinoma cell lines. Luciferase assays confirmed that the expression level of the wild type, but not the mutant plasmid, was significantly repressed in miR-22 mimic cells (Figures 3(c) and 3(d)). Next, we evaluated the expression level of NET1 mRNA and protein in the cultivated cells via using QRT-PCR and western blotting. Our data showed that NET1 mRNA level dropped less than 50% after the transfection of miR-22 mimic compared to the nc-mimic cells (Figure 3(e)). Similar findings were also observed in the protein level of the two cells (Figure 3(f)). All these results demonstrated that NET1 is directly regulated by miR-22.

### 3.3. miR-22 Controls Cellular Processes of NSCLC

**Figure 4:**
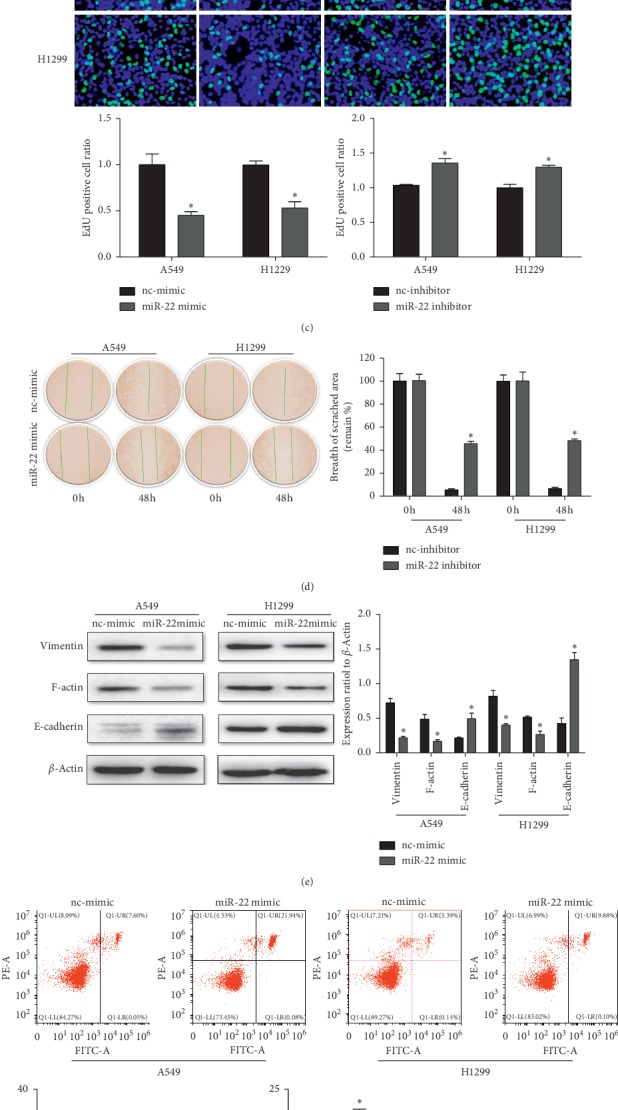
miR-22 weakens the proliferation and migration of NSCLC cells and enhances cell apoptosis. (a) Quantitative reverse transcription polymerase chain reactions (qRT-PCRs) showed that miR-22 mimics and inhibitors successfully take effect in both A549 and H1299 cells. (b) CCK-8 assay indicated that miR-22 mimics decreased cell's vitality, while miR-22 inhibitors increased cell's vitality (*n* = 3). (c) Similarly, EdU staining confirmed that miRNA-22 mimics resulted in a reduced cell's proliferation, while miR-22 inhibitors increased that effect (*n* = 4). (d) miR-22 mimics attenuated NSCLC cell migration in A549 and H1299 cells (*n* = 3). (e) Western blot was used to analyze EMT, and *β*-actin was considered as a loading control (*n* = 3). (f) Induction of cell apoptosis confirmed by flow cytometry (*n* = 4). (g) Apoptosis associated protein was analyzed using western blot, and *β*-actin was used as a control (*n* = 3). ^*∗*^*P* < 0.05.

To test whether miR-22 played a functional role in cellular effects of NSCLC, we firstly transfected miR-22 mimic, inhibitor, or their negative controls into A549 and H1299 cell lines for 48 h. Using QRT-PCR, we further verified that miR-22 expression was notably increased by miR-22 mimic, while reduced by miR-22 inhibitor (Figure 4(a)), indicating that miR-22 mimic and inhibitor successfully modulated miR-22 expression level in A549 and H1299 cells.

As confirmed by CCK-8 and EdU cell proliferation assay, miR-22 mimic significantly increased cell viability and proliferation, respectively, while miR-22 inhibitor decreased those effects (Figures 4(b) and 4(c)). These data showed that upregulation of miR-22 promoted A549 and H1299 cell proliferation while downregulation of miR-22 suppressed A549 and H1299 cell proliferation.

To determine the role of miR-22 in NSCLC metastasis, we used wound healing assays to evaluate the migration capacity of two LUAD cell lines. Our result showed that the miR-22 mimic weakens cell migration in both A549 and H1299 cell lines (Figure 4(d)), and western blot further demonstrated that overexpression of miR-22 could decrease the expression of vimentin and F-actin while increase the expression of E-cadherin (Figure 4(e)). Collectively, our results manifested that miR-22 acted as an antineoplastic microRNA and contributed to inhibit NSCLC cell migration in vitro.

As enhanced cell proliferation is related to apoptotic resistance, flow cytometry was performed to evaluate the effects of miR-22 on progression of A549 and H1299 cell apoptosis. Our results revealed that the miR-22 mimic promoted the A549 and H1299 cell's apoptosis, but the miR-22 inhibitor had the opposite effects (Figure 4(f)). Simultaneously, the upregulation of miR-22 led to the lower expression of Bcl-2 and increased expression level of the Bax ratio at the protein levels, but suppression of miR-22 reversed that effects (Figure 4(g)). These results indicate the proapoptotic effect of miR-22 in A549 and H1299 cells.

### 3.4. miR-22 Reduces Proliferation, Migration, and Apoptosis Resistance by Modulating NET1

**Figure 5:**
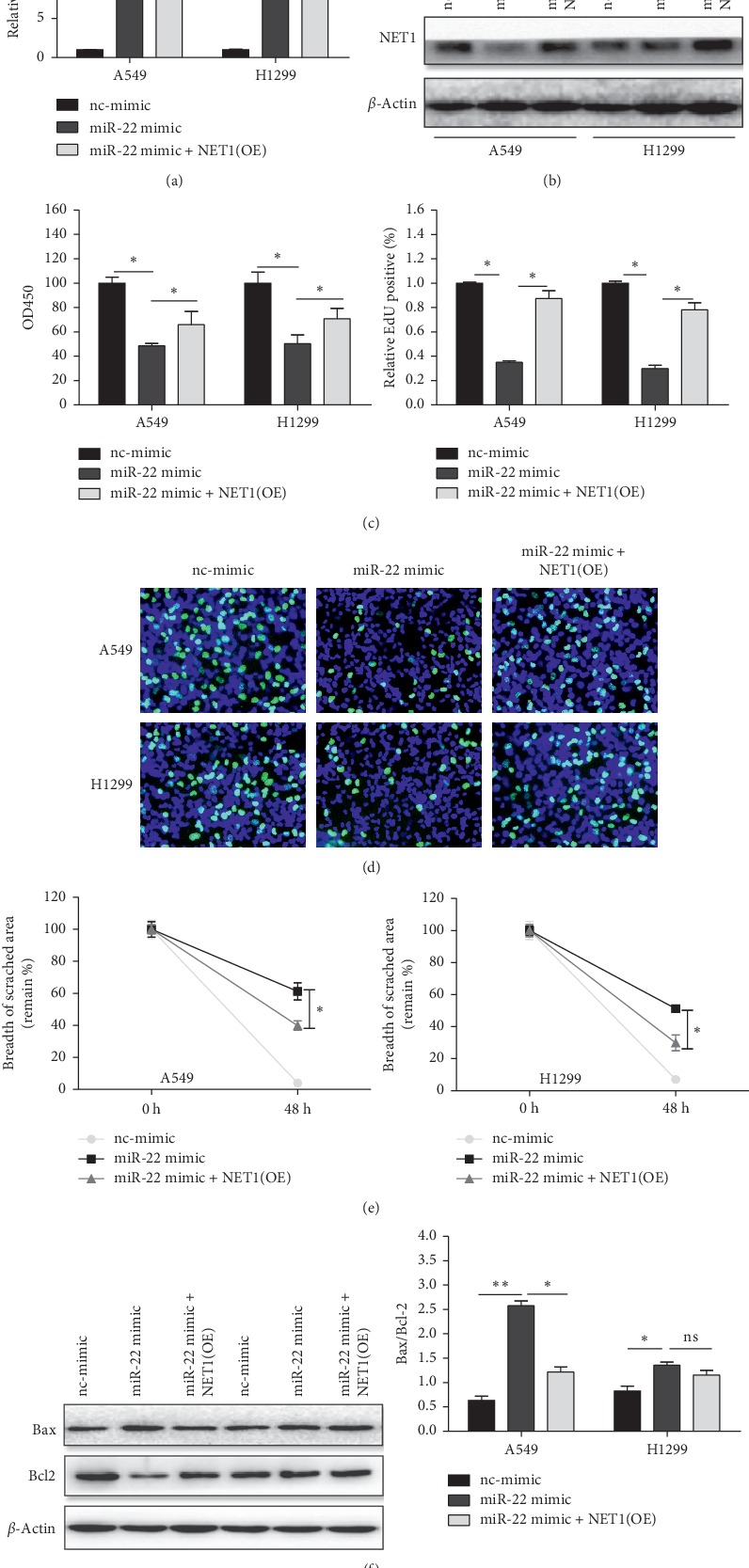
Overexpression of NET1 without 3′UTR rescued the roles of miR-22 in cell proliferation, migration, and apoptosis in NSCLC cells. (a, b) The expression level of miR-22 and its target gene NET1 was evaluated by using qRT-PCR and western blot, respectively (*n* = 3). (c, d) Overexpression of NET1 counteracted the inhibition of the cell vitality and growth capacity using CCK-8 and EdU assays (*n* = 4). (e) The suppression of migration of A549 and H1299 cells could be reversed by NET1 vector using wound healing assay (*n* = 3). (f) Analogously, NET1 vector neutralized the proapoptotic effect of miR-22 via using western blotting analyses (*n* = 3). ^*∗*^*P* < 0.05.

To verify if NET1 is closely related target of miR-22, we transduced A549 and H1299 cells with miR-22 mimic or its negative control in combination with overexpressed NET1 vector in NSCLC cells without the 3′UTR and validated the expression of miR-22 and NET1 (Figures 5(a) and 5(b)). Moreover, we surveyed the ability of cell proliferation, migration, and apoptosis. Hence, we found that the miR-22 attenuated proliferation and migration, and apoptotic resistance of NSCLC was reversed, at least partially, by the NET1 overexpression vector (Figures 5(c)–5(f)). These findings indicated that miR-22 weakens the growth, migration, and antiapoptotic effect of NSCLC cells via the miR-22/NET1 signaling pathway.

## 4. Discussion

Emergent evidence indicates that aberrant expression of miRNAs is a frequent event in the development and progression of lung cancer. The dysregulated miRNAs and their target genes have been identified to facilitate cell growth, metastasis, and apoptosis in human lung carcinomas.

miRNAs control a variety of essential cell biological processes and cellular pathways, and a single miRNA can negatively control multiple target genes [28]. Although the neuroepithelial cell transforming gene 1 (NET1) is identified as an oncogene in many human cancers [8, 11, 12, 14], including lung cancer [15], miRNAs that could directly modulate NET1 in the regulation of NSCLC cancer are poorly elucidated. For instance, downregulation of NET1 targeted by miR-22 has been reported to promote chemoresistance, and miR-22 acts as a novel prognostic biomarker in bladder cancer patients [26]. miR-22 is downregulated in chronic myeloid leukemia cells and is involved in CML cell growth and proliferation via targeting NET1 [27].

Based on an integrated database, dbDEMC (database of Differentially Expressed MiRNAs in human Cancers), we identified that miR-22 was reduced in human lung cancer. We also confirmed that miR-22 was downregulated in human NSCLC samples and cell lines compared with their normal controls using qRT-PCR. In line with our findings, miR-22 was much lower in lung cancer specimens and NSCLC cells than that in normal groups [24]. A shortcoming of our present work was the small sample size of lung cancer specimens and paired adjacent normal tissues. However, the keynote of our study was not only to identify differentially expressed miRNAs but also to reveal the clinical significance regarding functional roles in lung cancer cells. As predicted by the Targetscan and miRDB database, NET1 was a target gene of miR-22 in lung cancer. We emphasize to analyze the effect and function of miR-22 as well as its underlying potential target NET1 in lung cancer. Our observations indicated that miR-22 enhanced cell apoptosis and retarded both cell proliferation and migration, while inhibition of miR-22 had a contrary effect, indicating the latent growth-inhibiting and apoptosis-promoting effects of miR-22 in human lung cancer.

Neuroepithelial cell transforming gene 1 (NET1), a RhoA specific guanine nucleotide exchange factor, was first isolated from neuroepithelioma cells in 1996 [5]. NET1 plays a critical role in N-cadherin expression, cytoskeletal reorganization, and activation of RhoA(13). Therefore, overexpression of NET1 was closely related to malignant cellular biological behaviors [29] and was also associated with a variety of human cancers, including acute lymphoblastic leukemia [14], breast cancer [30], hepatocellular carcinoma [10], and non-small-cell lung cancer [15]. Consistently, bioinformatics validation, luciferase reporter assay, and western blotting analysis demonstrated that NET1 was directly and negatively targeted by miR-22 in both A549 and H1299 human NSCLC cell lines. Furthermore, assays of functional reconstruction revealed that a reduced NET1 expression was required to reverse the suppressive effect of miR-22 upregulation on cellular growth and migration and apoptotic resistance in NSCLC cells. We have previously reported that silencing NET1 can lead to the activation of RhoA signaling and thus attenuate cellular proliferation and migration during lung tumorogenesis [16]. Taken together, the present results clearly revealed that NET1 is a target gene of miR-22 in human NSCLC patients. Further study will be necessary to focus on the in vivo roles of miR-22 together with NET1 that appear to play a pivotal role in lung cancer.

In conclusion, the present study demonstrated that the lack of miR-22 is a common event in patients with NSCLC and may serve as a tumor suppressor by endogenously and negatively regulating NET1. To the best of our knowledge, we have identified, for the first time, that the miR-22/NET1 axis regulates the proliferation, migration, and apoptosis of NSCLC cells. These findings may provide a new evidence for better understanding the functional roles of miR-22 in the pathogenesis of NSCLC and developing new therapeutic strategy for NSCLC.

## Data Availability

The data and materials used during our present study are available from the corresponding author upon reasonable request.
